# Development and Validation of Deep-Learning-Based Sepsis and Septic Shock Early Prediction System (DeepSEPS) Using Real-World ICU Data

**DOI:** 10.3390/jcm12227156

**Published:** 2023-11-17

**Authors:** Taehwa Kim, Yunwon Tae, Hye Ju Yeo, Jin Ho Jang, Kyungjae Cho, Dongjoon Yoo, Yeha Lee, Sung-Ho Ahn, Younga Kim, Narae Lee, Woo Hyun Cho

**Affiliations:** 1 Division of Pulmonology, Allergy and Critical Care Medicine, Department of Internal Medicine, School of Medicine, Pusan National University and Research Institute for Convergence of Biomedical Science and Technology, Pusan National University Yangsan Hospital, Yangsan 50612, Republic of Korea; taehwagongju@naver.com (T.K.); dugpwn@naver.com (H.J.Y.); jjhteen1@naver.com (J.H.J.); 2 VUNO, Seoul 06541, Republic of Korea; yunwon.tae@vuno.co (Y.T.); kcho035@vuno.co (K.C.); dongjoon.yoo@vuno.co (D.Y.); yeha.lee@vuno.co (Y.L.); 3 Department of Internal Medicine, School of Medicine, Pusan National University, Busan 46241, Republic of Korea; 4 Department of Critical Care Medicine and Emergency Medicine, Inha University College of Medicine, Incheon 22212, Republic of Korea; 5 Division of Biostatistics, Department of Neurology, Research Institute for Convergence of Biomedical Science and Technology, Pusan National University Yangsan Hospital, Yangsan 50612, Republic of Korea; caesar-ahn@hanmail.net; 6 Department of Pediatrics, School of Medicine, Pusan National University, Yangsan 50612, Republic of Korea; youngflo@daum.net (Y.K.); nahrae111@gmail.com (N.L.)

**Keywords:** sepsis, sepsis-3, early prediction program, machine learning, artificial intelligence

## Abstract

Background: Successful sepsis treatment depends on early diagnosis. We aimed to develop and validate a system to predict sepsis and septic shock in real time using deep learning. Methods: Clinical data were retrospectively collected from electronic medical records (EMRs). Data from 2010 to 2019 were used as development data, and data from 2020 to 2021 were used as validation data. The collected EMRs consisted of eight vital signs, 13 laboratory data points, and three demographic information items. We validated the deep-learning-based sepsis and septic shock early prediction system (DeepSEPS) using the validation datasets and compared our system with other traditional early warning scoring systems, such as the national early warning score, sequential organ failure assessment (SOFA), and quick sequential organ failure assessment. Results: DeepSEPS achieved even higher area under receiver operating characteristic curve (AUROC) values (0.7888 and 0.8494 for sepsis and septic shock, respectively) than SOFA. The prediction performance of traditional scoring systems was enhanced because the early prediction time point was close to the onset time of sepsis; however, the DeepSEPS scoring system consistently outperformed all conventional scoring systems at all time points. Furthermore, at the time of onset of sepsis and septic shock, DeepSEPS showed the highest AUROC (0.9346). Conclusions: The sepsis and septic shock early warning system developed in this study exhibited a performance that is worth considering when predicting sepsis and septic shock compared to other traditional early warning scoring systems. DeepSEPS showed better performance than existing sepsis prediction programs. This novel real-time system that simultaneously predicts sepsis and septic shock requires further validation.

## 1. Introduction

The definition of sepsis has been revised over the past decades to detect its onset early and stratify disease severity [1,2]. Previous diagnostic criteria for sepsis and septic shock were inconvenient and complex, leading to delays in recognizing sepsis [3,4]. Recently, research has supported the early recognition of sepsis, resulting in reduced sepsis-related mortality [5,6,7,8]. In this context, efforts have been made for early recognition of sepsis and septic shock [9,10,11,12]. The third international consensus definition for sepsis and septic shock developed a simplified criteria for sepsis-related organ dysfunction and quick sequential organ failure assessment (qSOFA) [1]. The introduction of qSOFA is expected to be more accessible and practical for early screening of sepsis. However, the adoption of qSOFA, the new screening tool for sepsis, was not conducive to early sepsis screening and remains controversial [13,14,15]. Many studies have shown that qSOFA has a relatively high specificity for early detection of sepsis-related organ dysfunction [16,17,18,19].

In the absence of traditional diagnostic scales that can more clearly and quickly diagnose sepsis, artificial intelligence (AI) can potentially play a significant role in diagnosing sepsis. AI algorithms can be used to analyze large amounts of medical data, including patient information, laboratory results, and imaging studies to identify sepsis-related patterns and risk factors. AI automatically collects data according to set rules and processes. It also processes large amounts of data at a very high speed, so it can predict patterns and trends in the data. This process reduces errors due to subjective judgment, errors in data entry, and errors in data processing, resulting in high accuracy in data collection. This can help healthcare providers make faster and more accurate diagnoses, leading to early intervention and reduction of disease severity [20]. Additionally, AI can monitor patients with sepsis in real-time, alert healthcare providers to detect changes in a patient’s condition, and ensure timely intervention [20,21].

The use of AI in sepsis has continued to evolve [22], and it has the potential to revolutionize healthcare providers’ approach to this serious disease. Therefore, we aimed to develop a system to predict the new occurrence of sepsis and septic shock in real time using deep learning. Furthermore, we evaluate its performance in comparison with the conventional sepsis screening score system. This manuscript is written following the TRIPOD checklist [23].

## 2. Methods

### 2.1. Study Design and Setting

We retrospectively collected longitudinal clinical information of patients admitted to the intensive care unit (ICU) in a single tertiary center between 1 January 2010 and 31 December 2021. We used eight vital signs, 13 laboratory data points, and three demographic information items from the collected electronic medical record (EMR) records as variables. The baseline characteristics are summarized in Table 1 and Supplementary Table S1. We used the data from 2010 to 2019 as development data, and from 2020 to 2021 as validation data. The study was conducted in accordance with the Declaration of Helsinki (as revised in 2013). This study was approved by the Pusan National University Yangsan Hospital (PNUYH) Institutional Review Board (IRB No. 05-2021-291). The requirement for individual consent was waived due to the retrospective nature of the study.

### 2.2. Study Population

**Figure 1:**
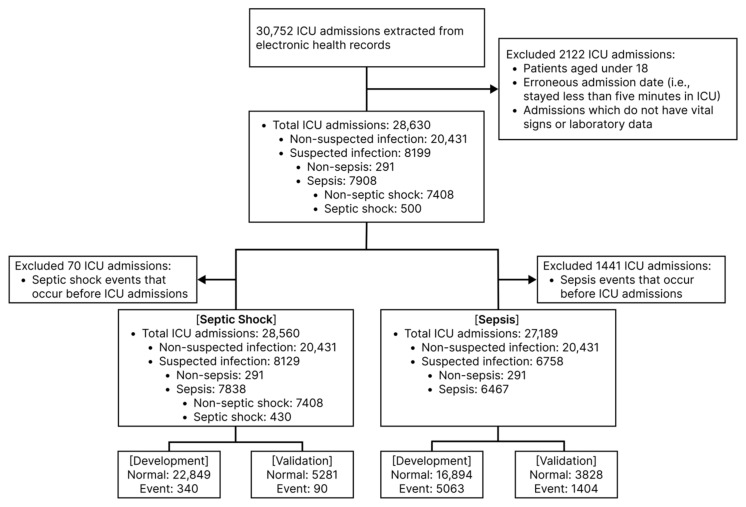
Study flow chart. ICU, intensive care unit.

**Figure 2:**
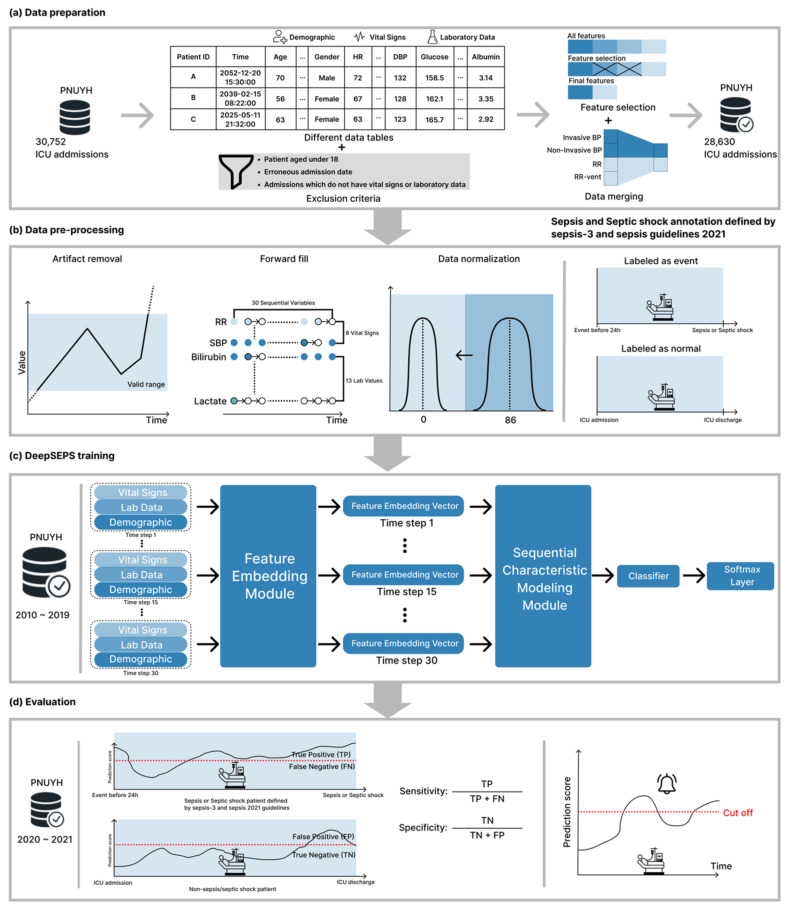
Process of development and validation overview. PNUYH, Pusan national university Yangsan hospital; ICU, intensive care unit; HR, heart rate; DBP, diastolic blood pressure; BP, blood pressure; RR, respiratory rate; SBP, systolic blood pressure; TP, true positive; FN, false negative; FP, false positive; TN, true negative.

The overall study flowchart is shown in Figure 1. A total of 28,630 patients were admitted to the medical or surgical ICU of the mentioned hospital during the study period (2010–2021). The patients aged 18 or less were excluded from the study. (Detailed data preparation procedure is depicted in Figure 2a) The sepsis or septic shock definition in all patients during the entire study period followed the Sepsis-3 diagnostic criteria. Prior to defining sepsis, suspected infection was defined as cases who received an antibiotic prescription after orders for blood cultures within 72 h or who received an order for blood culture following an antibiotic prescription within 24 h. (A detailed explanation of the operational definition can be found in Supplement Information; Supplementary S1). According to this operational definition, we identified 8199 patients with suspected infection and 7908 of them had an acute change in total sequential organ failure assessment (SOFA) score ≥ 2 points following the infection. This satisfied the Sepsis-3 guidelines and they were classified as having sepsis. Additionally, we identified 500 patients as positive for septic shock based on the following criteria: (1) hypotensive (less than 65 mm Hg mean arterial pressure (MAP)) after adequate fluid resuscitation (e.g., over 30 mL/kg of fluids), (2) greater than 2 mmol/L serum lactate, and (3) the need for a vasopressor to maintain a MAP greater than 65 mmHg [1] (Supplement Information; Supplementary S1).

### 2.3. Data Processing

All data was extracted in compliance with ethical standards and data security was maintained. Predefined rules and template models were used to extract and structure the collected data to ensure the accuracy of the material, all of which was done with two-factor encryption.

If variable lengths are different, we zero-padded the variables; thus, all input features can have the same lengths. Presumptive noisy records were treated as missing values using the following criteria: (1) Non-numeric records were considered missing values, and (2) Laboratory findings in the abnormal range (i.e., outlier measurements) were considered missing values. All variables were used for sepsis and septic shock risk score calculation whenever at least one input clinical feature was given, and the overall score was provided immediately. The valid ranges of each vital sign and laboratory value are presented in Supplementary Table S2. In addition to non-numeric or outlier measurements, missing values inevitably occurred because each vital sign and laboratory measurement were are recorded asynchronously, i.e., irregularly. As shown in Figure 2b, we imputed all the missing values by carrying forward immediate past values. However, we processed the missing values for the SOFA score differently because the score is measured in 24-h periods [24]. To obtain the total SOFA score, we must evaluate six organ systems: the respiratory, coagulation, liver, cardiovascular, renal, and neurological. Considering these six organ systems simultaneously is challenging because each SOFA score is recorded irregularly. Therefore, we utilized medical records from the past 24 h. Specifically, we present the most recent medical records. However, if records did not exist in the past 24 h, we assigned a zero score for the concerned organ system. We considered the records up to 48 h before ICU admissions to reduce the zero score of six organ systems. Through this process, we tried to reduce errors due to missing data. (A comparison of using and not using 48-h prior ICU data is provided in Supplementary S2 Section S1 and Supplementary Table S3.) The deep learning-based sepsis and septic shock early prediction system (DeepSEPS) complied with the comprehensive recommendation guideline set of the prediction system development [23,25].

### 2.4. Development and Training of DeepSEPS

As illustrated in Figure 2c, DeepSEPS consists of three modules: feature embedding, sequential characteristic modeling, and a classifier. The feature-embedding module, including eight fully connected networks (FCN) with normalization layers, allows the system to learn each input feature. The sequential characteristic modelling module comprises two gated recurrent unit networks [26] that capture sequential relationships. The system utilizes up to the most recent 30 sequentially input features (eight vital signs and 13 laboratory results can be entered several times) for analysis and sequential outputs the result. The input illustration is depicted in Figure 2b. Then, the last time-step representation was passed to the classifier module. The classifier module is a one-layer FCN followed by a softmax layer that returns a score between zero and one. Furthermore, we applied dropout [27], a regularization technique, to each neural network to prevent overfitting of the training data.

### 2.5. Evaluation

Using clinical records, DeepSEPS provides the prediction score of the occurrence of sepsis or septic shock in all patients who may or may not develop sepsis or septic shock. We used probability scores to compute the evaluation metrics, area under the receiver operating characteristic curve (AUROC), precision-recall curve (AUPRC), Youden’s index, sensitivity, specificity, positive predictive values (PPVs), negative predictive values (NPVs), and F-measures, and compared conventional system. The overall evaluation procedure is depicted in Figure 2d evaluation section.

### 2.6. Statistical Analysis

Statistical difference between development and validation is evaluated using Welch’s *t*-test and chi-square test for continuous and categorical variables respectively. These statistical tests are computed in Python 3.8.5 using sklearn v0.24. A statistical significance test was conducted through DeLong’s test (*p* < 0.001) in R v3.6.3 (http://www.r-project.org, accessed on 12 September 2023). For computing the confidence interval, we utilized 1000 bootstraps.

## 3. Results

### 3.1. Performance of DeepSEPS for Sepsis and Septic Shock

DeepSEPS used the development data collected during the period 2010–2019. We utilized the Adam optimizer [28] to update the neural network parameters by minimizing cross-entropy loss. We leveraged the development data from 2019 to tune the hyperparameters, such as the number of neural network layers and the probability of dropout. Additionally, we iteratively trained DeepSEPS and selected the best training moment, i.e., the one that provided the highest AUROC score for development data from 2019. The overall development and validation processes are illustrated in Supplementary Figure S1.

**Figure 3:**
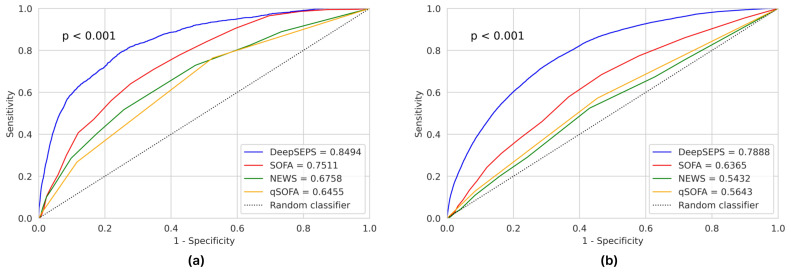
Area under receiver operating characteristic curves (AUROCs) for (**a**) septic shock and (**b**) sepsis prediction. DeepSEPS, deep-learning-based sepsis and septic shock early prediction system; SOFA, sequential organ failure assessment; NEWS, national early warning score; qSOFA, quick sequential organ failure assessment.

We validated DeepSEPS using validation datasets and compared it with other traditional early warning score systems, such as National Early Warning Score (NEWS), SOFA, and qSOFA, as in previous studies [29,30,31]. We used two metrics to evaluate each scoring system: AUROC and AUPRC. As shown in Table 2, DeepSEPS outperformed all other scoring systems in predicting the occurrence of new sepsis or septic shock. We conducted the DeLong’s test (*p* < 0.001) to demonstrate that DeepSEPS was significantly different than the other methods. In the validation dataset, SOFA, which had the highest AUROC among the conventional scoring systems, achieved AUROC values of 0.6365 (95% confidence interval (CI), 0.6325–0.6403) and 0.7511 (95% CI, 0.7407–0.7615); however, DeepSEPS achieved an even higher AUROC, 0.7888 (95% CI, 0.7855–0.7918) and 0.8494 (95% CI, 0.8423–0.8560) for sepsis and septic shock, respectively. The receiver operating characteristic curve (ROC) plot is shown in Figure 3. Moreover, in Supplementary Tables S4–S8, we compared the conventional scoring systems with the same specificity cutoff for 24-h early prediction. Although ROC curve plots provided an overview of the prediction performance, analyzing the sensitivity of a particular cutoff can be laborious. Therefore, we presented all the sensitivity and specificity results for the conventional scoring systems with the same specificity cutoff values used for DeepSEPS. The results demonstrated that DeepSEPS exhibited a higher sensitivity and F-measure than the other methods at the same specificity cutoff. We also illustrated these results qualitatively using a patient example in Supplementary Figure S1.

### 3.2. Results of Sensitivity and Specificity

As outlined in Supplementary Table S9, we computed the Youden’s index, sensitivity, specificity, PPVs, NPVs, and F-measures for various cutoff values for predicting septic shock and sepsis. Cutoff values of 30 and 35 for predicting septic shock and sepsis, respectively, yielded the highest Youden’s index. At these cutoff values, the sensitivities were 0.7985 and 0.7681, whereas the specificities were 0.7416 and 0.6633, respectively. For increased cutoff values of 95 for septic shock and 75 for sepsis, DeepSEPS achieved the highest F-measure scores of 0.0738 and 0.285, PPVs of 0.0539 and 0.2229, and NPVs of 0.9975 and 0.9573, respectively. The low PPVs were because of a class imbalance between event and normal patients. However, Supplementary Tables S4–S6 show that the PPVs were relatively higher than those obtained using other conventional scoring systems.

### 3.3. Performance Change of DeepSEPS and Other Systems According to the Time Interval from the Onset of Sepsis and Septic Shock

**Figure 4:**
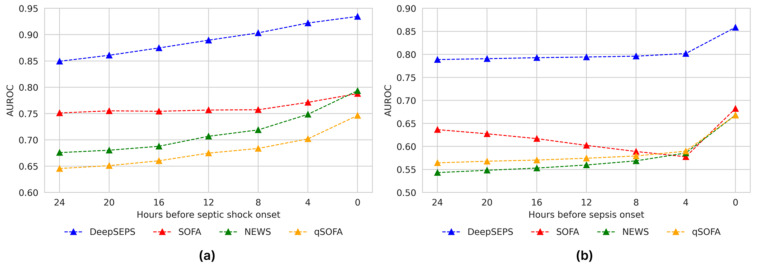
Prediction results for (**a**) septic shock and (**b**) sepsis based on different prediction time windows. DeepSEPS, deep-learning-based sepsis and septic shock early prediction system; SOFA, sequential organ failure assessment; NEWS, national early warning score; qSOFA, quick sequential organ failure assessment.

Although DeepSEPS was developed to predict sepsis and septic shock events 24 h in advance, it is important to show consistent predictions for all time windows. As shown in Figure 4, each scoring system is evaluated using different prediction time windows. Each conventional scoring system monotonically increased the AUROC scores as the early prediction time window approached the onset time. These systems showed the highest AUROC scores at the time of onset; however, their prediction performance was lower than that of the 24 h early prediction for DeepSEPS. In other words, even if we utilized a scoring system to screen for sepsis or septic shock events at the onset time, its screening performance was lower than the 24-h early prediction for DeepSEPS. Additionally, DeepSEPS enhanced the AUROC scores as onset approached. It achieved the highest AUROC scores at the onset time, which were 0.9346 and 0.8587 for septic shock and sepsis, respectively.

### 3.4. Comparison of Alarm Rates between DeepSEPS and Conventional Scoring Systems

**Figure 5:**
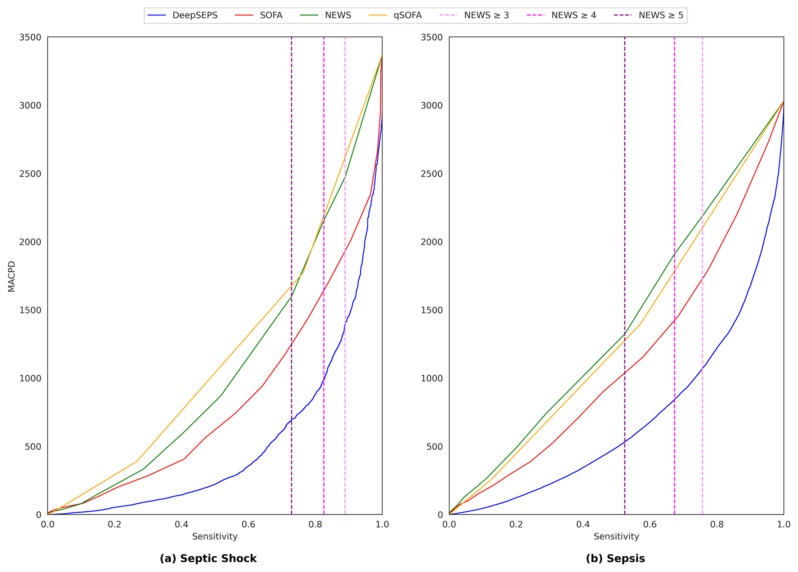
Mean alarm counts per day (MACPD) with respect to sensitivity for both (**a**) septic shock and (**b**) Sepsis 24-h early prediction. MACPD represents the number of alarms per day per 100 patients. For example, 300 MACPD indicates that if there are 100 patients in the intensive care unit (ICU), an alarm is triggered 300 times a day. DeepSEPS, deep-learning-based sepsis and septic shock early prediction system; SOFA, sequential organ failure assessment; NEWS, national early warning score; qSOFA, quick sequential organ failure assessment.

The number of alarms encountered by clinicians is a significant factor, because an excessive number of alarms may cause alarm fatigue, leading to important clinical events being missed [32,33,34]. We provided results regarding the mean alarm counts per day (MACPD), which represents the number of alarms per day per 100 patients. For example, 300 MACPD indicates that if there are 100 patients in the ICU, an alarm is triggered 300 times a day. Figure 5 shows the overall MACPD with respect to the sensitivity. DeepSEPS consistently exhibited a low MACPD, even with the same sensitivity for both sepsis and septic shock 24-h early prediction.

### 3.5. Interpretation of DeepSEPS

**Figure 6:**
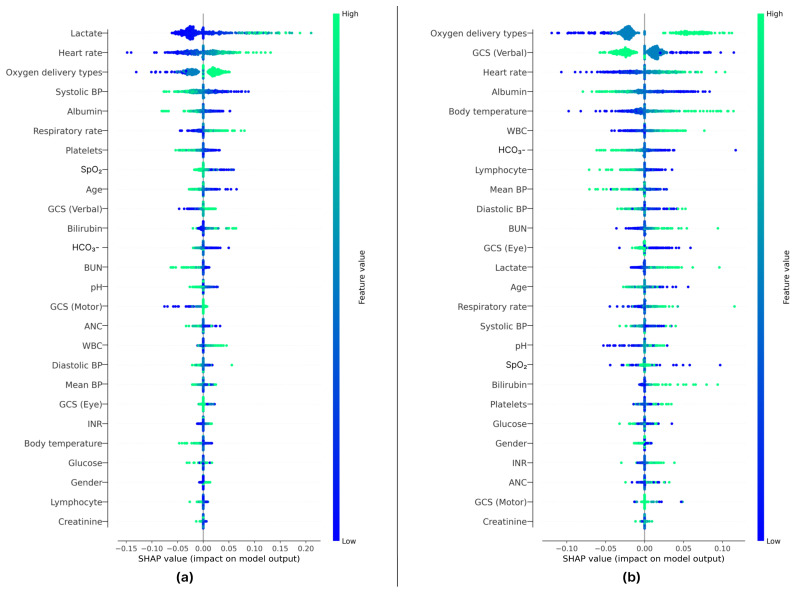
Feature importance of 500 random samples of vital signs and laboratory data from patients. The results of the septic shock and sepsis predictions are depicted in (**a**,**b**), respectively, where the most contributing features are arranged from top to bottom. The color of each dot represents the feature values and the x-axis shows the Shapley values. If multiple dots overlapped, they were displaced along the y-axis. Negative and positive Shapley values indicate whether a feature contributes to the production of low- or high-risk scores, respectively. For instance, in (**a**), if patients provided low lactate values (i.e., blue dots), the model was less likely to predict septic shock. However, if patients yield high lactate values (i.e., green dots), the model was more likely to classify them as having septic shock. BP, blood pressure; SpO_2_, oxygen saturation; GCS, Glasgow Coma Scale; WBC, white blood cell; pH, percentage of hydrogen ions; HCO_3_^−^, bicarbonate; BUN, blood urea nitrogen; INR, international normalized ratio; ANC, absolute neutrophil count.

Although our deep learning-based early prediction system exhibited promising results in septic shock and sepsis prediction, it may be difficult to interpret the results compared with those of conventional scoring systems. Hence, in this experiment, we used the Shapley Additive Explanations (SHAP) [35] to interpret DeepSEPS. As shown in Figure 6, 500 vital signs, laboratory data, and demographics were randomly selected. Figure 6a,b depict the septic shock and sepsis prediction results, respectively. Each dot represents a randomly selected patient at a specific time during the ICU stay, where blue indicates a high feature value and green indicates a low feature value. Negative and positive Shapley values represent the likelihood that the sample contributes to the model outputs. If the dot is blue and provides negative Shapley values (e.g., the lactate feature in Figure 6a), a low feature value makes the model less likely to classify patients as having septic shock. The variables most strongly associated with sepsis were lactate, heart rate, and oxygen delivery types, while variables of lesser association were gender, lymphocyte, and creatinine. DeepSEPS depended most heavily on lactate in predicting septic shock. Lactate contributed more to the prediction of septic shock than to the prediction of sepsis. Additionally, in predicting sepsis, the model primarily relied on information about the type of oxygen delivery, which was categorized into room air (blue), noninvasive ventilation (green-blue), and invasive ventilation (green).

### 3.6. Effectiveness of Glasgow Coma Scale (GCS) in Deep-Learning-Based Sepsis and Septic Shock Early Prediction System (DeepSEPS)

**Figure 7:**
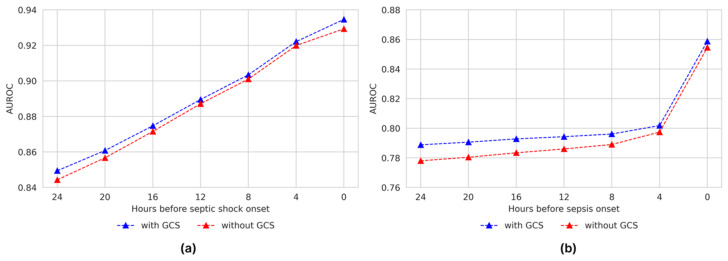
Effectiveness of Glasgow Coma Scale (GCS) with different prediction time windows. The left plot (**a**) demonstrates the septic shock prediction results, while the right plot (**b**) shows the sepsis prediction results. AUROC, under receiver operating characteristic curves.

Conventional scoring systems such as the sequential organ failure assessment (SOFA) and national early warning score (NEWS) require consciousness assessment scales (e.g., GCS and Alert, Verbal, Pain, and Unresponsive (AVPU) scales) to evaluate neurological systems. However, acquiring consciousness scales from ICU patients is challenging because they are often sedated. In Supplementary Table S10 and Figure 7, we show the effectiveness of the GCS input feature for DeepSEPS by comparing two different systems, with and without GCS. We evaluated the predictive results for both septic shock and sepsis. The evaluation results demonstrated performance degradation for both sepsis and septic shock events. However, considering the accessibility of collecting consciousness scales in the ICU, these performance drops are acceptable because DeepSEPS, without the GCS, still outperforms other traditional scoring systems.

### 3.7. Comparison of DeepSEPS and Other Machine Learning Models

As shown in Table 3, we provided an additional comparison of other machine learning models, Random Forest and Transformer. These two models were often used in sepsis prediction [36,37,38,39]. To be specific, we utilized the Random Forest classifier from sklearn v0.24. For the Transformer baseline, we changed the sequential characteristic module of DeepSEPS to the Transformer. The experimental results reveal the superior performance of DeepSEPS in comparison to recent machine learning models. For sepsis prediction, DeepSEPS exhibited an AUROC of 0.7888 (95% CI: 0.7855–0.7918), outperforming Transformer (AUROC: 0.7771, 95% CI: 0.7739–0.7804) and Random Forest (AUROC: 0.7064, 95% CI: 0.7028–0.7103). Moreover, in the task of septic shock prediction, DeepSEPS demonstrated a robust AUROC of 0.8494 (95% CI: 0.8423–0.856), surpassing Transformer (AUROC: 0.8147, 95% CI: 0.8067–0.8228) and Random Forest (AUROC: 0.7765, 95% CI: 0.7696–0.7842). The AUPRC score presented similar results compared with the AUROC score, in that DeepSEPS still outperformed Random Forest and Transformer. Overall, these results underscore the effectiveness of deep learning, particularly DeepSEPS, in achieving superior discrimination ability for early prediction of sepsis and septic shock when compared to recent machine learning counterparts.

## 4. Discussion

We developed and validated a deep learning-based prediction system for the occurrence of sepsis and septic shock. DeepSEPS not only predicted the occurrence of sepsis and septic shock early but also showed a low rate of false alarms. DeepSEPS can analyze both the events simultaneously, demonstrating acceptable performance. Additionally, this system can predict the onset of sepsis or septic shock in real-time.

As studies have shown that the early recognition of sepsis significantly affects clinical outcomes, many attempts have been made to screen and detect sepsis early. Sepsis-3 guidelines were published based on the evidence regarding the effects of early diagnosis and management. However, contradictory data have revealed that the qSOFA from the Sepsis-3 guidelines does not lead to early detection of sepsis [40,41]. Thus, a deep learning-based approach was proposed as an alternative tool. The use of AI in sepsis has continued to evolve since then [22], and it has the potential to revolutionize healthcare providers’ approach to this serious disease. Many researchers have developed sepsis alert systems using deep learning methods. These models show fair prediction of sepsis or septic shock; however, some drawbacks have been reported. Overfitting is a significant problem caused by training-biased datasets when developing an AI prediction model using supervised deep learning [42]. Previous study has limited sepsis cases in which cultures were proven [43]. This is more specific but can lead to undesirably biased results. The performance of sepsis and septic shock prediction systems depends on the accuracy and sensitivity of early recognition of events [44,45]. The sensitivity and accuracy of recognizing these events may vary, depending on the definition of sepsis. Because sepsis is a syndrome, each manifestation must be characterized to reflect the heterogeneity of its definition. In this study, sepsis was defined according to current guidelines using the concept of suspected infection. Our dataset also contained information on fluid resuscitation, allowing a clear definition of septic shock.

Generally, clinical data on ward patients are limited in amount and fragmented in time. Developing an AI algorithm predicting sepsis in real-time in advance from those data with loose time intervals is hard. In this context, we chose an ICU cohort and collected 21 significant variables with tight time intervals. Septic shock develops quickly and without apparent signs in patients admitted to an ICU, often leaving medical staff with insufficient time for life-saving interventions. Sometimes early detection is difficult given the non-specific nature of the initial symptoms. Effective screening tools can help address this problem by helping clinicians focus on patients at a high risk of septic shock. In this study, DeepSEPS exhibited a higher ROC for predicting for the occurrence of septic shock than conventional scoring systems, such as SOFA, NEWS, and qSOFA (AUROC = 0.8494, Figure 3a). Particularly, it showed consistently high predictive power in all time periods from 24 h before the onset of septic shock to the time of shock, and the predictive power further increased as the time of shock onset drew closer (AUROC = 0.9346, Figure 4a). The conventional scoring system also showed the highest predictive power at the time of occurrence; however, its performance declined as it moved back from the onset of septic shock. Moreover, we provided an additional experiment regarding the robustness of the Glasgow Coma Scale (GCS) input feature on DeepSEPS since ICU patients are often sedated. As elaborated in Section 3.6, DeepSEPS, without the GCS input feature, still performed better than other conventional scoring systems, SOFA and NEWS that require consciousness assessment scales. Also for AUPRC, DeepSEPS outperformed NEWS, SOFA, qSOFA, Random Forest, and Transformer. This result shows that DeepSEPS could better predict sepsis and septic shock.

This study’s limitations include the homogeneity of the training dataset and the absence of external validation of the developed system. An external validation or large prospective clinical study is essential to confirm the performance of AI prediction system. For instance, Supplementary S2 Section S2 in Supplementary Figure S2 and Table S11 showed that the lactate testing rate increases over time. Although we provided the performance of DeepSEPS on development and validations in Supplementary Figure S3 and attempted to reflect the recent clinical practice by evaluating DeepSEPS in recent years of the validation set, external validation and large prospective clinical studies are still essential to confirm the performance and prove the efficacy of DeepSEPS. To improve the performance and prove efficacy of DeepSEPS, we have a plan to conduct a prospective clinical trial. In the future study, we will conduct a multi-center clinical studies to investigate whether DeepSEPS improves clinical outcomes.

Additionally, the PPV of DeepSEPS was low. Mostly, low PPV is a common issue when the prevalence of the event is very low in dataset. This issue was also seen in other previous studies predicting sepsis onset [46,47]. To overcome this, we optimized the features used in the model, tried several different prediction models, and used stringent criteria to improve the accuracy of data labelling. Moreover, we also provided the comparison with other recent machine learning approaches, e.g., Transformer and Random Forest based sepsis prediction models [36,37,38,39]. As illustrated in Table 3, DeepSEPS still showed the comparable performance.

Despite the limitations, DeepSEPS has certain advantages. DeepSEPS is a dual prediction system that simultaneously predicts the occurrence of new sepsis or septic shock in ICU patients. Compared with previous systems that only predicted sepsis or septic shock, DeepSEPS could predict both events simultaneously and showed acceptable AUROC at a point. Notably, the possibility of prediction bias was reduced by making the definition of sepsis similar to that in a clinical scenario. The right interaction between clinicians and AI systems is key to the success of these technologies in becoming embedded in healthcare workflows. Seamless interaction ensures that AI systems provide accurate information and that clinicians avoid over-relying on technology, while clinician’s judgment and contextual understanding can still play an important role.

## 5. Conclusions

Deep learning-based event prediction systems are being increasingly utilized in critical care. DeepSEPS is a deep learning-based clinical decision-supporting system that predicts the onset of sepsis and septic shock in real-time. Although AI models cannot replace clinical expertise, they can help improve sepsis outcomes through early detection and timely management, in conjunction with clinician judgment. This can be achieved through large-scale clinical trials and ongoing monitoring of algorithm performance in real-world settings.

## Figures and Tables

**Table 1:** Summary of baseline characteristics for positive and negative event patients.

Baseline Characteristics	Sepsis Negative	Sepsis Positive	*p*-Value	Shock Negative	Shock Positive	*p*-Value
Number of admissions (%)	20,722 (76.21%)	6467 (23.79%)	-	28,130 (98.49%)	430 (1.51%)	-
per 1000 admissions	762.15	237.85	-	984.94	15.06	-
Number of vital signs & laboratory values (%)	1,529,125 (93.57%)	105,157 (6.43%)	-	5,335,227 (99.74%)	14,095 (0.26%)	-
Gender (%)	20,722 (100%)	6467 (100%)	*p* < 0.001	28,130 (100%)	430 (100%)	*p* = 0.03
Male	12,450 (60.08%)	4085 (63.17%)	-	17,148 (60.96%)	240 (55.81%)	-
Female	8272 (39.92%)	2382 (36.83%)	-	10,982 (39.04%)	190 (44.19%)	-
Age (mean ± SD)	62.98 ± 14.1	63.52 ± 14.48	*p* < 0.001	62.99 ± 14.1	61.03 ± 14.11	*p* < 0.001
Number of oxygen delivery types in admissions (%)	42,058 (100%)	6759 (100%)	*p* < 0.001	42,212 (100%)	511 (100%)	*p* < 0.001
Room air	7859 (18.69%)	465 (6.88%)	-	7923 (18.77%)	10 (1.95%)	-
Non-invasive ventilation	25,452 (60.52%)	3311 (48.99%)	-	25,518 (60.45%)	154 (30.14%)	-
Invasive ventilation	8747 (20.79%)	2983 (44.13%)	-	8771 (20.78%)	347 (67.91%)	-
8 vital signs (mean ± SD)	-	-	-	-	-	-
Heart rate (/min)	83.47 ± 19.93	92.53 ± 21.04	*p* < 0.001	89.32 ± 20.51	106.83 ± 25.04	*p* < 0.001
Diastolic blood pressure (mm Hg)	67.1 ± 14.98	66.96 ± 15.40	*p* = 0.01	66.5 ± 14.87	61.06 ± 16.33	*p* < 0.001
Systolic blood pressure (mm Hg)	122.63 ± 22.44	122.88 ± 24.48	*p* = 0.004	122.61 ± 23.43	105.06 ± 26.49	*p* < 0.001
Mean blood pressure (mm Hg)	83.09 ± 17.45	83.34 ± 17.49	*p* < 0.001	83.2 ± 35.38	74.48 ± 18.69	*p* < 0.001
Respiratory rate (/min)	18.2 ± 5.49	18.98 ± 6.38	*p* < 0.001	19.02 ± 7.18	20.69 ± 8.27	*p* < 0.001
Body temperature (℃)	36.57 ± 0.55	36.68 ± 0.54	*p* < 0.001	36.64 ± 0.68	36.61 ± 0.6	*p* < 0.001
SpO_2_ (%)	98.02 ± 5.04	97.95 ± 4.43	*p* < 0.001	98.24 ± 4.69	96.33 ± 7.4	*p* < 0.001
Total GCS	13.86 ± 1.53	13.09 ± 2.19	*p* < 0.001	13.51 ± 1.86	13.29 ± 2.34	*p* = 0.034
13 laboratory data (mean ± SD)	-	-	-	-	-	-
Lactate (mmol/L)	2.36 ± 2.74	2.49 ± 2.5	*p* = 0.013	2.26 ± 2.5	6.14 ± 4.69	*p* < 0.001
Bilirubin (mg/dL)	1.28 ± 2.21	2.82 ± 6.24	*p* < 0.001	2.63 ± 5.34	4.54 ± 6.27	*p* < 0.001
Platelets (10^3^/µL)	180.83 ± 96.21	151.98 ± 103.79	*p* < 0.001	165.55 ± 118.17	100.82 ± 87.07	*p* < 0.001
Creatinine (mg/dL)	1.24 ± 2.76	1.61 ± 3.97	*p* < 0.001	1.30 ± 2.8	1.50 ± 3.22	*p* = 0.21
WBC (10^3^/µL)	10.82 ± 7.35	11.91 ± 6.99	*p* < 0.001	11.08 ± 6.98	11.80 ± 9.4	*p* = 0.083
pH	7.42 ± 0.08	7.42 ± 0.09	*p* < 0.001	7.43 ± 0.08	7.37 ± 0.11	*p* < 0.001
HCO_3_^−^ (mmol/L)	24.20 ± 5.4	23.87 ± 5.71	*p* < 0.001	24.87 ± 5.62	21.67 ± 6.28	*p* < 0.001
BUN (mg/dL)	21.17 ± 17.87	27.74 ± 22.5	*p* < 0.001	27.26 ± 21.02	30.63 ± 25.07	*p* = 0.007
Albumin (g/dL)	3.25 ± 0.53	3.00 ± 0.53	*p* < 0.001	3.07 ± 0.55	2.69 ± 0.57	*p* < 0.001
Glucose (mg/dL)	159.93 ± 68.96	172.86 ± 76.24	*p* < 0.001	170.61 ± 75.31	168.13 ± 89.05	*p* = 0.373
INR	1.35 ± 0.59	1.51 ± 0.72	*p* < 0.001	1.42 ± 0.58	1.88 ± 1.21	*p* < 0.001
Lymphocyte (10^3^/µL)	1.14 ± 0.73	0.94 ± 0.63	*p* < 0.001	1.02 ± 0.78	0.97 ± 1.4	*p* = 0.406
ANC	8720.00 ± 5359.11	9955.29 ± 6311.88	*p* < 0.001	9000.29 ± 5673.99	9838.68 ± 8625.27	*p* = 0.031

We conduct Welch’s *t*-test for continuous variables and Chi-square test for categorical variables to provide *p*-values and show the difference between normal and event patients. SpO_2_, oxygen saturation; GCS, Glasgow Coma Scale; WBC, white blood cell; pH, percentage of hydrogen ions; HCO_3_^−^, bicarbonate; BUN, blood urea nitrogen; INR, international normalized ratio; ANC, absolute neutrophil count; SD, standard deviation.

**Table 2:** Comparison of deep-learning-based sepsis and septic shock early prediction system (DeepSEPS) and other scoring systems on septic shock and sepsis predictions.

Metric	Target Event	DeepSEPS (95% CI)	SOFA (95% CI)	qSOFA (95% CI)	NEWS (95% CI)
AUROC	Sepsis	0.7888 (0.7855–0.7918)	0.6365 (0.6325–0.6403)	0.5643 (0.5609–0.5677)	0.5432 (0.5392–0.5473)
	Septic shock	0.8494 (0.8423–0.856)	0.7511 (0.7407–0.7615)	0.6455 (0.6356–0.655)	0.6758 (0.6665–0.6858)
AUPRC	Sepsis	0.2289 (0.2236–0.2346)	0.0943 (0.0926–0.0965)	0.084 (0.0817–0.0865)	0.0706 (0.0693–0.0721)
	Septic shock	0.0317 (0.0274–0.0372)	0.0075 (0.0070–0.0078)	0.0167 (0.0135–0.02)	0.0063 (0.0059–0.0068)

AUROC, area under receiver operating characteristic curve; AUPRC, area under precision-recall curve; CI: confidence interval; SOFA, sequential organ failure assessment; qSOFA, quick sequential organ failure assessment; NEWS, national early warning score.

**Table 3:** Comparison of deep-learning-based sepsis and septic shock early prediction system (DeepSEPS) and other machine learning models, Transformer and Random Forest, on septic shock and sepsis predictions.

Metric	Target Event	DeepSEPS (95% CI)	Transformer (95% CI)	Random Forest (95% CI)
AUROC	Sepsis	0.7888 (0.7855–0.7918)	0.7771 (0.7739–0.7804)	0.7064 (0.7028–0.7103)
	Septic shock	0.8494 (0.8423–0.856)	0.8147 (0.8067–0.8228)	0.7765 (0.7696–0.7842)
AUPRC	Sepsis	0.2289 (0.2236–0.2346)	0.2171 (0.2114–0.2228)	0.1331 (0.1302–0.1402)
	Septic shock	0.0317 (0.0274–0.0372)	0.0173 (0.0158–0.019)	0.0107 (0.0097–0.0118)

AUROC, area under receiver operating characteristic curve; AUPRC, area under precision-recall curve; CI, confidence interval.

## Data Availability

The datasets used and/or analyzed during the current study are available from the corresponding author on reasonable request.

## Supplementary Materials

The following supporting information can be downloaded at: https://www.mdpi.com/article/10.3390/jcm12227156/s1, Table S1: Summary of baseline characteristics for development and validation data; Table S2: Definition of valid range for each vital sign and laboratory values; Table S3: Difference between intensive care unit (ICU) admission and sepsis onset times; Table S4: Comparison between deep-learning-based sepsis and septic shock early prediction system (DeepSEPS) and quick sequential organ failure assessment (qSOFA) for equal specificities; Table S5: Comparison between deep-learning-based sepsis and septic shock early prediction system (DeepSEPS) and national early warning score (NEWS) for equal specificities; Table S6: Comparison between deep-learning-based sepsis and septic shock early prediction system (DeepSEPS) and sequential organ failure assessment (SOFA) for equal specificities; Table S7: Comparison between deep-learning-based sepsis and septic shock early prediction system (DeepSEPS) and national early warning score (NEWS) for equal specificities; Table S8: Comparison between deep-learning-based sepsis and septic shock early prediction system (DeepSEPS) and sequential organ failure assessment (SOFA) for equal specificities; Table S9: Classification results of deep-learning-based sepsis and septic shock early prediction system (DeepSEPS) for different cutoff values; Table S10: Effectiveness of glasgow coma scale (GCS) in deep-learning-based sepsis and septic shock early prediction system (DeepSEPS); Table S11: Lactate testing rates; Figure S1: Trajectory of features and risk scores for one patient; Figure S2: Lactate testing rates by year; Figure S3: Prediction results for (a) septic shock and (b) sepsis between development and validation datasets. References [1,48] are cited in the Supplementary Materials.
