# Supplementary material for: Development and Validation of Deep-Learning-Based Sepsis and Septic Shock Early Prediction System (DeepSEPS) Using Real-World ICU Data
Source: J Clin Med. 2023 Nov 17;12(22):7156. doi: 10.3390/jcm12227156 (PMC10672000; doi:10.3390/jcm12227156)
Supplement: Supplementary file 1 [file jcm-12-07156-s001.zip › jcm-2696238-supplementary.pdf]

Development and validation of deep-learning-based sepsis and septic shock early prediction system (DeepSEPS) using real-world ICU data

List of contents

Supplementary S1 ……………………………………………………………………………………….…………….......2

Supplementary S2………………………………………………………………………..……………......3

Table S1. Summary of baseline characteristics for development and validation data ……………..……….…..3

Table S2. Definition of valid range for each vital sign and laboratory values ………..…………….…..……4

Table S3. Difference between intensive care unit (ICU) admission and sepsis onset times ……………...5

Table S4. Comparison between deep-learning-based sepsis and septic shock early prediction system (DeepSEPS) and quick sequential organ failure assessment (qSOFA) for equal specificities …………..5

Table S5. Comparison between deep-learning-based sepsis and septic shock early prediction system (DeepSEPS) and national early warning score (NEWS) for equal specificities ………………….………..5

Table S6. Comparison between deep-learning-based sepsis and septic shock early prediction system (DeepSEPS) and sequential organ failure assessment (SOFA) for equal specificities ……….…………...5

Table S7. Comparison between deep-learning-based sepsis and septic shock early prediction system (DeepSEPS) and national early warning score (NEWS) for equal specificities ..…………………………...6

Table S8. Comparison between deep-learning-based sepsis and septic shock early prediction system (DeepSEPS) and sequential organ failure assessment (SOFA) for equal specificities ………..……..…7

Table S9. Classification results of deep-learning-based sepsis and septic shock early prediction system (DeepSEPS) for different cutoff values ……………………………………………………………..…..9

Table S10. Effectiveness of Glasgow Coma Scale (GCS) in deep-learning-based sepsis and septic shock early prediction system (DeepSEPS)……………………………………………………………..…9

Table S11. Lactate testing rates ……………………………………………………….……….…....10

Figure S1. Trajectory of features and risk scores for one patient …………………………..…………10

Figure S2. Lactate testing rates by year………………………………………………………………10

Figure S3. Prediction results for (a) septic shock and (b) sepsis between development and validation datasets.

……………………………………………………………………………………………………11

Supplementary S1

Detailed explanation of the operational definition for sepsis and septic shock

1. The Sepsis-3 criteria for the diagnosis of sepsis and septic shock

Sepsis-3 was described in the 2016 consensus definition as a pattern of life-threatening organ dysfunction caused by a dysregulated host response to infection [1].

Septic shock was defined as persistent hypotension requiring vasopressors to maintain a mean arterial pressure (MAP) greater than 65 mm Hg and elevated serum lactate greater than 2 mmol/L, despite adequate fluid resuscitation (30 mL/kg of fluids, urine output > 0.5 mL/kg/h, CVP of 8–12 mm Hg) [1].

2. Suspected infection time definition

Suspected infection (SI) time was defined as the time at which antibiotics were administered or a blood culture test was performed.

1) Time of suspected infection if the culture test was performed within 24 h of antibiotic administration

2) If antibiotics were administered within 72 h of culture testing, it was defined as the time of suspected infection.

The earliest suspected infection time point of 1 or 2 was chosen.

3. Sepsis onset time definition

Sepsis onset time was defined as a sequential organ failure assessment (SOFA) score increase of 2 points in the past 48 h or in the future 24 h, based on the SI time. An interval of increase of 2 points or more in the SOFA score was defined as a difference of 2 points or more in the minimum SOFA score within 24 h. This definition is based on the Sepsis-3 standards.

4. Septic shock onset time definition

If all Steps 1–4 below are satisfied from the point of sepsis onset to the end of discharge, Step 1 was defined as the point of septic shock onset.

1) Step 1: Use a vasopressor.

2) Step 2: If the MAP dropped below 65 mm Hg within the range of the time of vasopressor use within the period of intensive care unit (ICU) admission in the past.

3) Step 3: If the fluid was provided at a total of 30 mL/kg or more for 3 h within 6 h, 3 h before, and after the use of the vasopressor, but a vasopressor was needed.

4) Step 4: If lactate exceeds two within a total of 48 h, 24 h before and after vasopressor use.

5. SOFA and quick sequential organ failure assessment (qSOFA) definition

Although the change in SOFA score is a robust mortality stratification tool, it is cumbersome to calculate, and requires laboratory values that are not readily available for the quick screening of patients outside the ICU. For example, the serum lactate level that is routinely analyzed from a blood gas sample in the ICU may be difficult to measure in a ward patient and on a serial basis. The task force was set out to identify readily accessible screening measures and arrive at three criteria termed the quick sequential organ failure assessment (qSOFA). For patients outside the ICU who met two or more of the following criteria: Glasgow score less than 13, systolic blood pressure less than 100 mm Hg, or respiratory rate (RR) ≥22, mortality was similar to that of patients identified using the full SOFA score.

6. National early warning score (NEWS) definition

The NEWS is a tool developed to detect and respond to clinical deterioration in adult patients based on a simple aggregate scoring system in which a score is allocated to physiological measurements (RR, oxygen saturation, systolic blood pressure, pulse rate, level of consciousness, and temperature). NEWS2 was published and updated in December 2017.

7. Code

Sepsis was confirmed based on the assignment of Korean Standard Classification of Diseases (KCD) codes for sepsis (KCD, 7th Edition code A41 series) and septic shock (KCD, 7th Edition code R57.2) at the ICU admission time. KCD codes are the same as International Classification of Diseases (ICD) 9 codes of codes 995.92 (severe sepsis) or 785.52 (septic shock).

Supplementary S2

Additional Experimental Results

Section S1: Effectiveness of utilizing 48-hour prior intensive care unit (ICU) data to define sepsis onset times

Developing a superior early prediction system requires accurate event onset times because it is trained or evaluated based on its defined onset times. In this study, we use 48-hour prior ICU data to increase the accuracy of sepsis onset times, as defined by the Sepsis-3 and Sepsis Guidelines 2021. Supplementary Table 4 reports the median time difference between ICU admission and sepsis onset within the interquartile range (IQR). Without using 48-hour prior ICU data (i.e., using only ICU data), defining sepsis onset times lags five hours compared to onset times labeled with 48-hour prior ICU data. This demonstrates that, despite the development of an early prediction system, actual sepsis and septic shock screening can be delayed by at least five hours.

Section S2: Lactate testing rates change over time

Sepsis guidelines have changed over time, which is directly reflected in retrospective clinical data. For instance, Supplementary Figure 6 shows that the lactate testing rate increases monotonically with time. In 2010, the lactate testing rate was 2.9%; however, in 2021, it increased to 63.67%. The lactate testing rates for the medical information mart for intensive care (MIMIC) datasets are shown in Supplementary Table 12. MIIMC-III was collected during 2001–2012, and MIMIC-IV was extracted during 2008–2019. Because the MIMIC datasets de-identify the years, we could only show the total testing rates. Considering that MIMIC-IV is more recent than MIMIC-III, the lactate testing rates were different: 65.06% in MIMIC-IV and 47.47% in MIMIC-III. Therefore, to correctly evaluate our system, we validated DeepSEPS from 2020 to 2021, which exhibited the highest lactate testing rates.

Table S1. Summary of baseline characteristics for development and validation data.

Baseline characteristics	Development (2010 ~ 2019)	Validation (2020 ~ 2021)	*p*-value
**Number of admissions (%)**	23,189 (81.19%)	5,371 (18.81%)	-
**Number of sepsis admissions (%)**	5,063 (78.29%)	1,404 (21.71%)	-
Prevalence	23.06%	26.83%	-
**Number of septic shock admissions (%)**	340 (79.07%)	90 (20.93%)	-
Prevalence	1.47%	1.68%	-
**Number of vital signs & laboratory values (%)**	5,688,676 (85.64%)	953,657 (14.36%)	-
**Gender (%)**	28,560 (100%)	5,371 (100%)	$p = 0.025$
Male	17,388 (60.88%)	3,357 (62.5%)	-
Female	11,172 (39.12%)	2,014 (37.5%)	-
**Age (mean ± SD)**	62.99 ± 14.1	64.32 ± 13.76	$p < 0.001$
**Number of oxygen delivery types in admissions (%)**	42,234 (100%)	8,030 (100%)	$p = 0.039$
Room air	7,927 (18.77%)	1,601 (19.94%)	-
Non-invasive ventilation	25,534 (60.46%)	4,759 (59.27%)	-
Invasive ventilation	8,773 (20.77%)	1,670 (20.8%)	-
**8 vital signs (mean ± SD)**	-	-	-
Heart rate (/min)	89.82 ± 20.66	88.05 ± 20.78	$p < 0.001$

Diastolic blood pressure (mm Hg)	66.41 ± 14.88	64.06 ± 14.52	$p < 0.001$
Systolic blood pressure (mm Hg)	122.21 ± 23.58	121.63 ± 23.46	$p < 0.001$
Mean blood pressure (mm Hg)	83.09 ± 34.57	82.56 ± 74.93	$p < 0.001$
Respiratory rate (/min)	19.05 ± 7.2	18.72 ± 5.87	$p < 0.001$
Body temperature (°C)	36.63 ± 0.67	36.56 ± 0.57	$p < 0.001$
SpO_2 (%)	98.23 ± 4.85	98.22 ± 5.98	$p = 0.283$
Total GCS	13.51 ± 1.86	13.38 ± 2.04	$p < 0.001$
**13 laboratory data (mean ± SD)**	-	-	-
Lactate (mmol/L)	2.33 ± 2.59	2.12 ± 2.3	$p < 0.001$
Bilirubin (mg/dL)	2.78 ± 5.55	2.93 ± 5.78	$p < 0.001$
Platelets (10^3/μL)	162.47 ± 117.96	156.58 ± 118.38	$p < 0.001$
Creatinine (mg/dL)	1.29 ± 2.83	1.30 ± 2.93	$p = 0.701$
WBC (10^3/μL)	11.13 ± 7.04	10.77 ± 5.83	$p < 0.001$
pH	7.43 ± 0.08	7.43 ± 0.08	$p = 0.353$
HCO_3- (mmol/L)	24.86 ± 5.65	26.06 ± 5.43	$p < 0.001$
BUN (mg/dL)	27.36 ± 20.96	26.73 ± 20	$p < 0.001$
Albumin (g/dL)	3.06 ± 0.55	3.02 ± 0.49	$p < 0.001$
Glucose (mg/dL)	170.34 ± 75.32	164.59 ± 76.35	$p < 0.001$
INR	1.42 ± 0.59	1.35 ± 0.52	$p < 0.001$
Lymphocyte (10^3/μL)	1.01 ± 0.78	0.94 ± 0.72	$p < 0.001$
ANC	9063.18 ± 5777.76	8807.06 ± 5370.74	$p < 0.001$

We conduct Welch's t-test for continuous variables and Chi-square test for categorical variables to provide *p*-values and show the difference between normal and event patient. SpO_2, oxygen saturation; GCS, Glasgow Coma Scale; WBC, white blood cell; pH, percentage of hydrogen ions; HCO_3-, bicarbonate; BUN, blood urea nitrogen; INR, international normalized ratio; ANC, absolute neutrophil count; SD, standard deviation.

Table S2. Definition of valid range for each vital sign and laboratory values.

**Input features**	**Valid ranges**
Heart rate (/min)	0 ~ 390
Diastolic blood pressure (mm Hg)	0 ~ 375
Systolic blood pressure (mm Hg)	0 ~ 375
Mean blood pressure (mm Hg)	0 ~ 375
Respiratory rate (/min)	0 ~ 330
Body temperature (°C)	14.2 ~ 47
SpO_2 (%)	All
Total GCS	3 ~ 15
Lactate (mmol/L)	0 ~ 33
Bilirubin (mg/dL)	0 ~ 66
Platelets (10^3/μL)	0 ~ 2200
Creatinine (mg/dL)	0 ~ 66
WBC (10^3/μL)	0 ~ 1100
pH	6.3 ~ 10
HCO_3- (mmol/L)	0 ~ 66
BUN (mg/dL)	0 ~ 275
Albumin (g/dL)	0 ~ 60
Glucose (mg/dL)	0 ~ 2200
INR	0 ~ 8
Lymphocyte (10^3/μL)	All
ANC	All

We refer the valid ranges provided from [48]. SpO_2, oxygen saturation; GCS, Glasgow Coma Scale; WBC, white blood cell; pH, percentage of hydrogen ions; HCO_3-, bicarbonate; BUN, blood urea nitrogen; INR, international normalized ratio; ANC, absolute neutrophil count.

Table S3. Difference between intensive care unit (ICU) admission and sepsis onset times.

**Data**	**Hours Before Sepsis Onset Median (IQR)**

with 48-hour prior ICU data	1.42 (0 – 52.63)
without 48-hour prior ICU data	6.72 (0.73 – 54.37)

Table S4. Comparison between deep-learning-based sepsis and septic shock early prediction system (DeepSEPS) and quick sequential organ failure assessment (qSOFA) for equal specificities.

Score System	Sensitivity	Specificity	PPV	NPV	F-measure
		**Septic Shock**			
**DeepSEPS ≥ 7.27**	**0.9351**	**0.4746**	**0.005**	**0.9996**	0.01
qSOFA ≥ 1	0.7635	0.4746	0.0041	0.9986	0.0082
DeepSEPS ≥ 63.13	0.6194	0.8844	0.015	0.9988	0.0293
qSOFA ≥ 2	0.2676	0.8844	0.0065	0.9976	0.0128
DeepSEPS ≥ 94.2	0.1492	0.9919	0.0499	0.9976	0.0748
qSOFA ≥ 3	0.0228	0.9919	0.008	0.9972	0.0118
		**Sepsis**			
DeepSEPS ≥ 22.37	0.8626	0.5461	0.1128	0.9835	0.1995
qSOFA ≥ 1	0.5721	0.5461	0.0778	0.9502	0.1369
DeepSEPS ≥ 76.69	0.3747	0.9161	0.2302	0.9563	0.2852
qSOFA ≥ 2	0.1285	0.9161	0.093	0.9402	0.1079
DeepSEPS ≥ 95.35	0.0616	0.9953	0.4702	0.9407	0.1087
qSOFA ≥ 3	0.008	0.9953	0.1014	0.9375	0.0147

PPV, positive predictive value; NPV, negative predictive value.

Table S5. Comparison between deep-learning-based sepsis and septic shock early prediction system (DeepSEPS) and national early warning score (NEWS) for equal specificities.

Score System	Sensitivity	Specificity	PPV	NPV	F-measure
		**Septic Shock**			
**DeepSEPS ≥ 9.53**	**0.9201**	**0.5265**	**0.0055**	**0.9996**	0.0109
NEWS ≥ 5	0.7288	0.5265	0.0044	0.9985	0.0087
DeepSEPS ≥ 68	0.5891	0.902	0.0168	0.9987	0.0326
NEWS ≥ 8	0.2857	0.902	0.0082	0.9978	0.016
DeepSEPS ≥ 95.29	0.1074	0.9949	0.057	0.9975	0.0746
NEWS ≥ 12	0.0091	0.9949	0.005	0.9972	0.0065
		**Sepsis**			
DeepSEPS ≥ 24.67	0.8469	0.5697	0.1164	0.9823	0.2046
NEWS ≥ 5	0.5245	0.5697	0.0754	0.9471	0.1319
DeepSEPS ≥ 76.67	0.3752	0.916	0.2302	0.9564	0.2853
NEWS ≥ 8	0.1092	0.916	0.0801	0.9389	0.0924
DeepSEPS ≥ 94.78	0.0755	0.9939	0.455	0.9414	0.1295
NEWS ≥ 12	0.0024	0.9939	0.0256	0.9371	0.0044

PPV, positive predictive value; NPV, negative predictive value.

Table S6. Comparison between deep-learning-based sepsis and septic shock early prediction system (DeepSEPS) and sequential organ failure assessment (SOFA) for equal specificities.

Score System	Sensitivity	Specificity	PPV	NPV	F-measure
		**Septic Shock**			
DeepSEPS ≥ 1.45	0.9886	0.2086	0.0035	0.9998	0.0071
SOFA ≥ 3	0.9858	0.2086	0.0035	0.9998	0.007
DeepSEPS ≥ 26.87	0.817	0.722	0.0083	0.9993	0.0164
SOFA ≥ 9	0.6411	0.722	0.0065	0.9986	0.0129
DeepSEPS ≥ 85.47	0.3743	0.9621	0.0274	0.9982	0.051
SOFA ≥ 15	0.1492	0.9621	0.0111	0.9975	0.0206

			Sepsis		
DeepSEPS ≥ 12.67	0.9199	0.4196	0.0959	0.9874	0.1737
SOFA ≥ 3	0.7745	0.4196	0.082	0.9653	0.1483
DeepSEPS ≥ 69.5	0.4579	0.8801	0.2036	0.9604	0.2819
SOFA ≥ 9	0.2423	0.8801	0.1192	0.9455	0.1598
DeepSEPS ≥ 91.99	0.1326	0.9852	0.3757	0.9444	0.1962
SOFA ≥ 15	0.0172	0.9852	0.072	0.9374	0.0277

PPV, positive predictive value; NPV, negative predictive value.

Table S7. Comparison between deep-learning-based sepsis and septic shock early prediction system (DeepSEPS) and national early warning score (NEWS) for equal specificities.

Score System	Sensitivity	Specificity	PPV	NPV	F-measure
			**Septic Shock**		
DeepSEPS ≥ 0.04	1	0	0.0028	0	0.0057
NEWS ≥ 1	1	0.0001	0.0028	1	0.0057
DeepSEPS ≥ 0.04	1	0	0.0028	0	0.0057
NEWS ≥ 2	1	0.0002	0.0028	1	0.0057
DeepSEPS ≥ 2.15	0.9791	0.2666	0.0038	0.9998	0.0075
NEWS ≥ 3	0.889	0.2666	0.0034	0.9988	0.0069
DeepSEPS ≥ 3.86	0.9563	0.3609	0.0042	0.9997	0.0084
NEWS ≥ 4	0.8253	0.3609	0.0037	0.9986	0.0073
DeepSEPS ≥ 9.53	0.9201	0.5265	0.0055	0.9996	0.0109
NEWS ≥ 5	0.7288	0.5265	0.0044	0.9985	0.0087
DeepSEPS ≥ 30.14	0.7973	0.7425	0.0087	0.9992	0.0173
NEWS ≥ 6	0.5175	0.7425	0.0057	0.9982	0.0113
DeepSEPS ≥ 47	0.6946	0.8241	0.0111	0.9989	0.0219
NEWS ≥ 7	0.4038	0.8241	0.0065	0.9979	0.0128
DeepSEPS ≥ 68	0.5891	0.902	0.0168	0.9987	0.0326
NEWS ≥ 8	0.2857	0.902	0.0082	0.9978	0.016
DeepSEPS ≥ 84	0.4065	0.957	0.0262	0.9982	0.0493
NEWS ≥ 9	0.1476	0.957	0.0097	0.9975	0.0182
DeepSEPS ≥ 89.91	0.2692	0.9769	0.0322	0.9979	0.0575
NEWS ≥ 10	0.0996	0.9769	0.0121	0.9974	0.0216
DeepSEPS ≥ 93.06	0.1806	0.988	0.0412	0.9976	0.0671
NEWS ≥ 11	0.0484	0.988	0.0114	0.9973	0.0185
DeepSEPS ≥ 95.29	0.1074	0.9949	0.057	0.9975	0.0746
NEWS ≥ 12	0.0091	0.9949	0.005	0.9972	0.0065
DeepSEPS ≥ 96.67	0.0563	0.9982	0.0833	0.9973	0.0672
NEWS ≥ 13	0.0039	0.9982	0.0061	0.9972	0.0048
DeepSEPS ≥ 97.5	0.0283	0.9994	0.1254	0.9972	0.0462
NEWS ≥ 14	0.0016	0.9994	0.0073	0.9972	0.0026
DeepSEPS ≥ 98.39	0.0094	0.9999	0.3529	0.9972	0.0184
NEWS ≥ 15	0	0.9999	0	0.9972	0
			**Sepsis**		
DeepSEPS ≥ 0.05	1	0	0.0627	0.9999	0.118
NEWS ≥ 1	0.9998	0.0001	0.0627	0.8696	0.118
DeepSEPS ≥ 0.05	1	0	0.0627	0.9999	0.118
NEWS ≥ 2	0.9996	0.0003	0.0627	0.9114	0.118
DeepSEPS ≥ 6.09	0.9626	0.28	0.0821	0.9911	0.1513
NEWS ≥ 3	0.7568	0.2805	0.0658	0.9452	0.121
DeepSEPS ≥ 10.05	0.9361	0.3729	0.0908	0.9887	0.1656
NEWS ≥ 4	0.6734	0.3729	0.067	0.9446	0.1219
DeepSEPS ≥ 24.67	0.8469	0.5697	0.1164	0.9823	0.2046
NEWS ≥ 5	0.5245	0.5697	0.0754	0.9471	0.1319

DeepSEPS ≥ 48.55	0.6602	0.7585	0.1547	0.9709	0.2506
NEWS ≥ 6	0.2899	0.7585	0.0744	0.9411	0.1184
DeepSEPS ≥ 62.25	0.5381	0.8403	0.184	0.9645	0.2743
NEWS ≥ 7	0.2014	0.8403	0.0778	0.9402	0.1123
DeepSEPS ≥ 76.67	0.3752	0.916	0.2302	0.9564	0.2853
NEWS ≥ 8	0.1092	0.916	0.0801	0.9389	0.0924
DeepSEPS ≥ 85.69	0.2403	0.9591	0.2825	0.9497	0.2597
NEWS ≥ 9	0.0434	0.9591	0.0663	0.9374	0.0524
DeepSEPS ≥ 90.29	0.1668	0.9787	0.3442	0.9461	0.2248
NEWS ≥ 10	0.0232	0.9787	0.0678	0.9374	0.0345
DeepSEPS ≥ 92.83	0.1171	0.9881	0.3977	0.9436	0.181
NEWS ≥ 11	0.0094	0.9881	0.0502	0.9371	0.0159
DeepSEPS ≥ 94.78	0.0755	0.9939	0.455	0.9414	0.1295
NEWS ≥ 12	0.0024	0.9939	0.0256	0.9371	0.0044
DeepSEPS ≥ 96.54	0.032	0.9979	0.5114	0.9391	0.0606
NEWS ≥ 13	0.0008	0.9979	0.0245	0.9372	0.0015
DeepSEPS ≥ 97.52	0.0161	0.9992	0.5857	0.9382	0.0314
NEWS ≥ 14	0.0002	0.9992	0.0183	0.9373	0.0004
DeepSEPS ≥ 98.33	0.0084	0.9998	0.79	0.9378	0.0166
NEWS ≥ 15	0.0001	0.9998	0.0339	0.9373	0.0002

PPV, positive predictive value; NPV, negative predictive value.

Table S8. Comparison between deep-learning-based sepsis and septic shock early prediction system (DeepSEPS) and sequential organ failure assessment (SOFA) for equal specificities.

Score System	Sensitivity	Specificity	PPV	NPV	F-measure
		**Septic Shock**			
DeepSEPS ≥ 0.26	1	0.0371	0.0029	1	0.0059
SOFA ≥ 1	0.9957	0.0371	0.0029	0.9997	0.0059
DeepSEPS ≥ 0.75	0.9996	0.1254	0.0032	1	0.0065
SOFA ≥ 2	0.9945	0.1254	0.0032	0.9999	0.0064
DeepSEPS ≥ 1.45	0.9886	0.2086	0.0035	0.9998	0.0071
SOFA ≥ 3	0.9858	0.2086	0.0035	0.9998	0.007
DeepSEPS ≥ 2.73	0.9721	0.3037	0.004	0.9997	0.0079
SOFA ≥ 4	0.965	0.3037	0.0039	0.9997	0.0078
DeepSEPS ≥ 4.97	0.9481	0.4053	0.0045	0.9996	0.009
SOFA ≥ 5	0.9052	0.4053	0.0043	0.9993	0.0086
DeepSEPS ≥ 8.04	0.9284	0.4939	0.0052	0.9996	0.0103
SOFA ≥ 6	0.8406	0.4939	0.0047	0.9991	0.0094
DeepSEPS ≥ 12.24	0.8957	0.5748	0.006	0.9995	0.0118
SOFA ≥ 7	0.7776	0.5748	0.0052	0.9989	0.0103
DeepSEPS ≥ 18.5	0.8548	0.6533	0.007	0.9994	0.0138
SOFA ≥ 8	0.7088	0.6533	0.0058	0.9987	0.0115
DeepSEPS ≥ 26.87	0.817	0.722	0.0083	0.9993	0.0164
SOFA ≥ 9	0.6411	0.722	0.0065	0.9986	0.0129
DeepSEPS ≥ 37.34	0.7517	0.7811	0.0097	0.9991	0.0191
SOFA ≥ 10	0.5604	0.7811	0.0072	0.9984	0.0143
DeepSEPS ≥ 49.49	0.6836	0.8341	0.0116	0.9989	0.0228
SOFA ≥ 11	0.4707	0.8341	0.008	0.9982	0.0158
DeepSEPS ≥ 62.04	0.6269	0.8806	0.0147	0.9988	0.0288
SOFA ≥ 12	0.4073	0.8806	0.0096	0.9981	0.0188
DeepSEPS ≥ 72.25	0.56	0.9163	0.0187	0.9986	0.0362
SOFA ≥ 13	0.2999	0.9163	0.0101	0.9978	0.0195
DeepSEPS ≥ 79.45	0.4782	0.9414	0.0227	0.9984	0.0433
SOFA ≥ 14	0.2074	0.9415	0.01	0.9976	0.0191
DeepSEPS ≥ 85.47	0.3743	0.9621	0.0274	0.9982	0.051
SOFA ≥ 15	0.1492	0.9621	0.0111	0.9975	0.0206
DeepSEPS ≥ 89.7	0.2743	0.9761	0.0317	0.9979	0.0568
SOFA ≥ 16	0.1047	0.9761	0.0123	0.9974	0.022

DeepSEPS ≥ 92.23	0.2011	0.9851	0.0371	0.9977	0.0626
SOFA ≥ 17	0.0417	0.9851	0.0079	0.9972	0.0133
DeepSEPS ≥ 94.17	0.1492	0.9918	0.0493	0.9976	0.0741
SOFA ≥ 18	0.0138	0.9918	0.0047	0.9972	0.0071
DeepSEPS ≥ 96.01	0.074	0.9967	0.0605	0.9974	0.0666
SOFA ≥ 19	0	0.9967	0	0.9972	0
DeepSEPS ≥ 96.99	0.0464	0.9988	0.1014	0.9973	0.0637
SOFA ≥ 20	0	0.9988	0	0.9972	0
DeepSEPS ≥ 97.86	0.0169	0.9997	0.1488	0.9972	0.0304
SOFA ≥ 21	0	0.9997	0	0.9972	0
DeepSEPS ≥ 98.39	0.0094	0.9999	0.3529	0.9972	0.0184
SOFA ≥ 22	0	0.9999	0	0.9972	0
		**Sepsis**			
DeepSEPS ≥ 1.8	0.9932	0.1027	0.069	0.9956	0.129
SOFA ≥ 1	0.9538	0.1027	0.0664	0.9708	0.1242
DeepSEPS ≥ 6.16	0.9622	0.282	0.0823	0.9911	0.1516
SOFA ≥ 2	0.8603	0.282	0.0742	0.9679	0.1367
DeepSEPS ≥ 12.67	0.9199	0.4196	0.0959	0.9874	0.1737
SOFA ≥ 3	0.7745	0.4196	0.082	0.9653	0.1483
DeepSEPS ≥ 21.22	0.87	0.5336	0.111	0.984	0.1968
SOFA ≥ 4	0.6844	0.5336	0.0894	0.9619	0.1582
DeepSEPS ≥ 31.38	0.7922	0.633	0.1262	0.9785	0.2178
SOFA ≥ 5	0.5785	0.633	0.0954	0.9573	0.1638
DeepSEPS ≥ 41.81	0.7165	0.7137	0.1435	0.9741	0.2391
SOFA ≥6	0.4604	0.7137	0.0971	0.9518	0.1604
DeepSEPS ≥ 51.74	0.631	0.7788	0.1603	0.9693	0.2556
SOFA ≥7	0.3824	0.7788	0.1037	0.9496	0.1631
DeepSEPS ≥ 61.63	0.5443	0.8369	0.1826	0.9648	0.2734
SOFA ≥8	0.3081	0.8369	0.1122	0.9476	0.1645
DeepSEPS ≥ 69.5	0.4579	0.8801	0.2036	0.9604	0.2819
SOFA ≥9	0.2423	0.8801	0.1192	0.9455	0.1598
DeepSEPS ≥ 75.45	0.3911	0.9099	0.2252	0.9571	0.2858
SOFA ≥10	0.1784	0.9099	0.117	0.943	0.1413
DeepSEPS ≥ 80.19	0.3273	0.9329	0.2462	0.954	0.281
SOFA ≥11	0.1335	0.9329	0.1175	0.9415	0.125
DeepSEPS ≥ 84.11	0.2676	0.9519	0.2714	0.951	0.2695
SOFA ≥12	0.0883	0.9519	0.1093	0.9398	0.0977
DeepSEPS ≥ 87.75	0.209	0.9681	0.3048	0.9482	0.248
SOFA ≥13	0.0569	0.9681	0.1066	0.9388	0.0742
DeepSEPS ≥ 89.9	0.1735	0.9773	0.3391	0.9464	0.2295
SOFA ≥14	0.0287	0.9773	0.0782	0.9377	0.042
DeepSEPS ≥ 91.99	0.1326	0.9852	0.3757	0.9444	0.1962
SOFA ≥15	0.0172	0.9852	0.072	0.9374	0.0277
DeepSEPS ≥ 93.73	0.0987	0.9911	0.427	0.9426	0.1603
SOFA ≥16	0.0098	0.9911	0.0681	0.9373	0.0171
DeepSEPS ≥ 94.7	0.0772	0.9937	0.4521	0.9415	0.132
SOFA ≥17	0.0064	0.9937	0.0635	0.9373	0.0117
DeepSEPS ≥ 95.38	0.0605	0.9954	0.4701	0.9406	0.1072
SOFA ≥18	0.0001	0.9954	0.0015	0.937	0.0002
DeepSEPS ≥ 96.22	0.0403	0.9973	0.5053	0.9395	0.0746
SOFA ≥19	0.0001	0.9973	0.0013	0.9371	0.0001
DeepSEPS ≥ 97.19	0.0216	0.9988	0.5563	0.9385	0.0414
SOFA ≥20	0	0.9988	0	0.9372	0
DeepSEPS ≥ 98.33	0.0084	0.9998	0.79	0.9378	0.0166
SOFA ≥21	0	0.9998	0	0.9373	0

PPV, positive predictive value; NPV, negative predictive value.

Table S9. Classification results of deep-learning-based sepsis and septic shock early prediction system (DeepSEPS) for different cutoff values.

Cutoff	Youden’s Index	Sensitivity	Specificity	PPV	NPV	F-measure
			**Septic Shock**			
5	0.3542	0.9477	0.4065	0.0045	0.9996	0.009
10	0.4502	0.9142	0.536	0.0056	0.9995	0.0111
15	0.494	0.8804	0.6136	0.0064	0.9994	0.0128
20	0.5148	0.8469	0.6679	0.0072	0.9993	0.0143
25	0.5349	0.8261	0.7089	0.008	0.9993	0.0159
30	0.5401	0.7985	0.7416	0.0087	0.9992	0.0173
35	0.5376	0.7682	0.7694	0.0094	0.9991	0.0186
40	0.5259	0.7324	0.7935	0.01	0.999	0.0197
45	0.5226	0.7068	0.8158	0.0108	0.999	0.0213
50	0.5174	0.6812	0.8361	0.0117	0.9989	0.023
55	0.5109	0.6556	0.8552	0.0127	0.9989	0.025
60	0.5105	0.6372	0.8734	0.0141	0.9988	0.0276
65	0.5003	0.6092	0.8911	0.0157	0.9988	0.0306
70	0.4878	0.5793	0.9085	0.0177	0.9987	0.0344
75	0.4513	0.5254	0.9259	0.0198	0.9985	0.0381
80	0.4132	0.4699	0.9433	0.023	0.9984	0.0439
85	0.3446	0.3841	0.9605	0.0269	0.9982	0.0503
90	0.2441	0.2668	0.9773	0.0323	0.9979	0.0577
95	0.111	0.1169	0.9942	0.0539	0.9975	0.0738
			**Sepsis**			
5	0.22	0.9729	0.2471	0.0796	0.9927	0.1471
10	0.3083	0.9364	0.3719	0.0907	0.9887	0.1654
15	0.3608	0.9055	0.4554	0.1001	0.9863	0.1803
20	0.3967	0.8766	0.5201	0.1089	0.9844	0.1938
25	0.4175	0.8446	0.573	0.1169	0.9822	0.2053
30	0.4236	0.8034	0.6202	0.124	0.9792	0.2148
35	0.4314	0.7681	0.6633	0.1324	0.9771	0.2259
40	0.431	0.7303	0.7007	0.1404	0.9749	0.2355
45	0.4247	0.6897	0.735	0.1483	0.9725	0.2442
50	0.4149	0.6471	0.7678	0.1572	0.9702	0.2529
55	0.4033	0.6046	0.7987	0.1673	0.9679	0.2621
60	0.3864	0.5588	0.8276	0.1782	0.9656	0.2703
65	0.3654	0.5099	0.8555	0.191	0.9631	0.2779
70	0.3351	0.4524	0.8827	0.2052	0.9601	0.2823
75	0.3032	0.3955	0.9077	0.2229	0.9573	0.2851
80	0.2622	0.3302	0.932	0.2452	0.9541	0.2814
85	0.2081	0.2521	0.956	0.2772	0.9503	0.264
90	0.1498	0.1721	0.9777	0.3407	0.9464	0.2286
95	0.0649	0.0705	0.9944	0.4592	0.9411	0.1222

PPV, positive predictive value; NPV, negative predictive value.

Table S10. Effectiveness of glasgow coma scale (GCS) in deep-learning-based sepsis and septic shock early prediction system (DeepSEPS).

Target Event	With GCS (95% CI)	Without GCS (95% CI)
Sepsis	0.7888<br>(0.7855-0.7918)	0.778<br>(0.7747-0.7814)
Septic shock	0.8494<br>(0.8423-0.856)	0.8443<br>(0.8367-0.8523)

CI, confidence interval.

Table S11. Lactate testing rates.

Data	Lactate Testing Rates (%)
MIMIC-III (2001 ~ 2012)	47.47%
MIMIC-IV (2008 ~ 2019)	65.06%

MIMIC, medical information mart for intensive care.

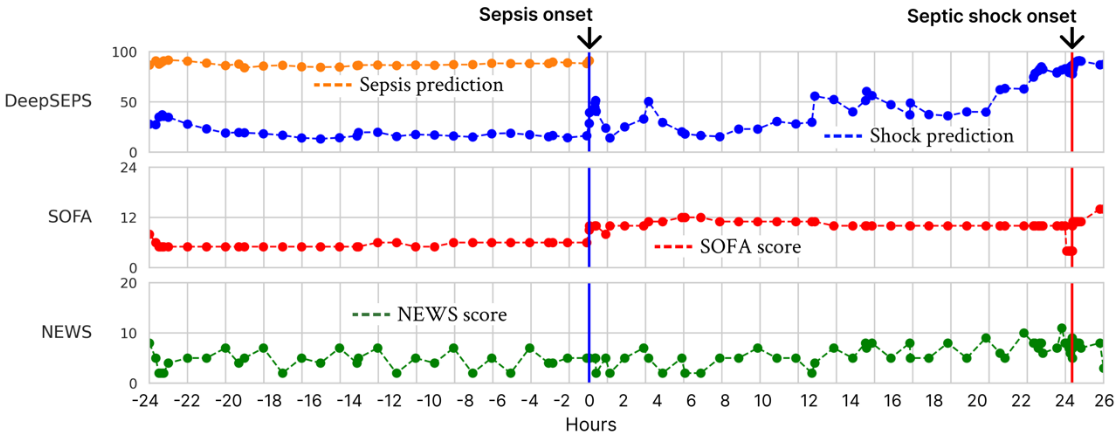

Figure S1 Trajectory of features and risk scores for one patient. The blue and red vertical lines indicate the sepsis and septic shock onset times defined by the Sepsis-3 and Sepsis Guidelines 2021, respectively. Deep-learning-based sepsis and septic shock early prediction system (DeepSEPS), sequential organ failure assessment (SOFA), and national early warning score (NEWS) represent the risk scores at each time point.

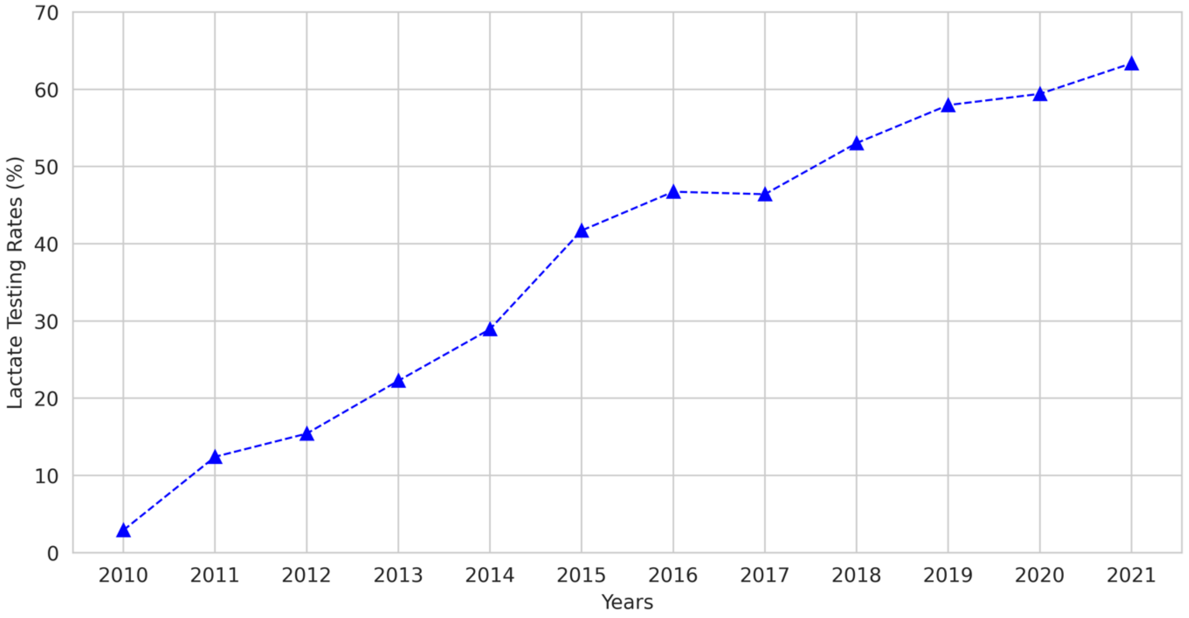

Figure S2. Lactate testing rates by year.

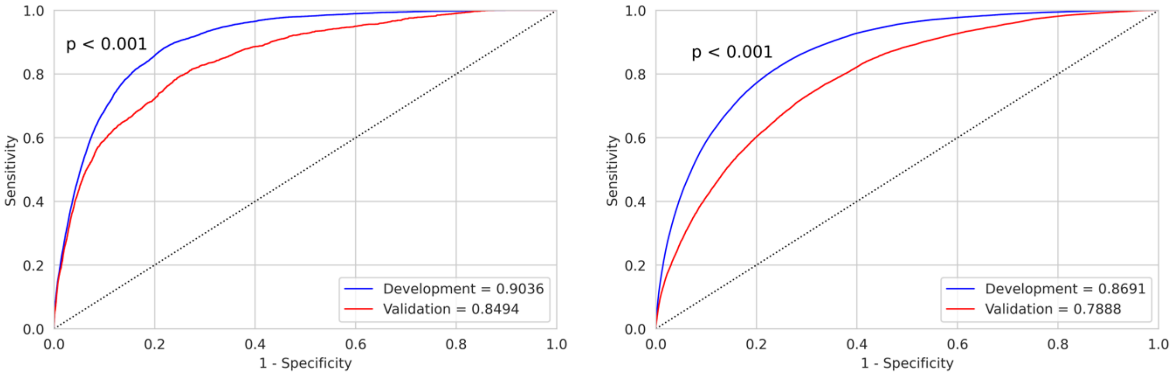

(a) (b)

Figure S3. Prediction results for (**a**) septic shock and (**b**) sepsis between development and validation datasets.
